# Alterations of DNA Methylation at GDNF Gene Promoter in the Ventral Tegmental Area of Adult Depression-Like Rats Induced by Maternal Deprivation

**DOI:** 10.3389/fpsyt.2018.00732

**Published:** 2019-01-10

**Authors:** Yi Zhang, Lei Wang, Xin Wang, Yuting Wang, Chuting Li, Xiongzhao Zhu

**Affiliations:** ^1^Medical Psychological Center, The Second Xiangya Hospital, Central South University, Changsha, China; ^2^Department of Clinical Psychology, Maternal and Child Health Hospital of Hunan Province, Changsha, China

**Keywords:** maternal deprivation, depression, GDNF, DNA methylation, ventral regimental area

## Abstract

**Objective:** To study the expression and DNA methylation of the Glial cell line-derived neurotrophic factor (GDNF) gene in the development of depression-like behaviors in rats experiencing maternal deprivation stress in early life.

**Methods:** Newborn SD rats were randomly assigned to a normal control group (NOR) or maternal deprivation group (MD). An open field test (OPT), sucrose preference test (SPT), and a forced swimming test (FST) were used to evaluate rats' behaviors. Protein, mRNA, and methylation levels were measured by ELISA/Western blot, real-time PCR, and BiSulfte Amplicon sequencing PCR, respectively.

**Results:** MD rats had significantly shorter total distance and more fecal pellets in OPT, a lower sucrose preference rate in SPT, and a longer immobility time in FST than NOR rats. Compared with NOR rats, MD rats showed a significantly higher plasma corticosterone (CORT) level. The levels of plasma dopamine (DA) and the GDNF were significantly lower in the MD rats than in NOR rats. In the ventral tegmental area (VTA) tissues, MD rats had a significantly higher level of methylation at the GDNF gene promoter than NOR rats. The expression of the GDNF mRNA and protein were significantly lower in MD rats than in NOR rats. The total distance was significantly correlated with plasma DA and GDNF, the DNA methylation level at the GDNF promoter and the GDNF mRNA level in the VTA. Fecal pellets showed a significant correlation with plasma CORT. The sucrose preference rate was significantly correlated with plasma DA, the DNA methylation level at the GDNF promoter and the GDNF mRNA level in the VTA. Immobility time showed a significant correlation with the plasma DA, the plasma GDNF and the GDNF mRNA level in the VTA.

**Conclusion:** up-regulation of DNA methylation at the GDNF gene promotor and the subsequent down-regulation of the GDNF gene expression in the VTA, may be involved in the development of depression-like behaviors in rats experiencing MD in early life.

## Introduction

Major depressive disorder (MDD) is a common psychiatric disorder with a lifetime prevalence of 3–17% (1). MDD is characterized by anhedonia, a depressive mood, and psychomotor retardation. Previous studies on the etiology, suggest that MDD is multifactorial and involves both genetic and environmental factors. Epidemiological studies demonstrated that early life stress is associated with the stress-related psychopathologies in later life, including depression (2, 3). However, the effect of early life adversity on depression is poorly understood, particularly, the influences of both the genetic and environmental factors.

The monoamine hypothesis proposes that MDD may be caused by the dysregulation of monoaminergic neurotransmitters in the central nervous system (CNS) including serotonin, dopamine (DA), and norepinephrine. Among them, dopamine is most abundant in the CNS and the body of dopaminergic neurons is mainly located in the ventral tegmental area (VTA) and projected to almost the entire brain (4), involved in the regulation of cognition, motivation, reward, reinforcement behavior, and emotions (5–7). Numerous studies have observed the deficiency of the dopaminergic system in patients with MDD (8–10). In addition, early life adversity influences the development of the dopaminergic system, followed by an impairment of its structures and functions (11–13).

The accumulated evidence revealed that the glial cell line-derived neurotrophic factor (GDNF) can promote the survival of dopamine neurons (14, 15). Kumar et al. (16) found that the GDNF can regulate the postnatal developments and adult functions of the dopamine system. It has been demonstrated that an exogenous increase of the GDNF level in the CNS, could increase the number of adult dopamine neurons or its terminals in the dorsal striatum. In addition, the GDNF can increase dopamine levels and augment dopamine release and re-uptake in the striatum (15). Moreover, the epigenetic status of the GDNF in the nucleus accumbens, is thought to be associated with the susceptibility and adaptation to chronic stressful events, and DNA hypermethylation in the promoter of the GDNF gene, which reduces the expression of the GDNF, and has been revealed to determine the behavioral responses to chronic stress (17).

To further investigate the epigenetic mechanisms of early life stress induced depression and to better characterize the role of the GDNF, maternal deprivation (MD), a well-known paradigm reflecting early life stress, was employed in this study to establish an animal model. The sucrose preference, open field, and forced swimming test were adopted to obtain behavioral data. The levels of CORT, DA, and the GDNF in plasma were measured, while the DNA methylation and expression of the GDNF gene in the VTA, were monitored to determine the relationship between biomarkers and the behavioral consequences.

## Materials and Methods

### Animal and Design

**Figure 1:**
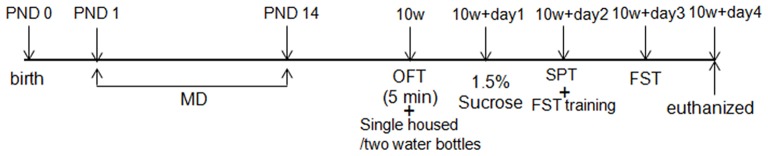
Experiment schedule. OFT, open-field test; SPT, sucrose preference test; FST, forced swimming test; PND, postnatal day; SD rats were assigned to two groups: the normal control group (NOR) and the maternal deprivation stress group (MD).

Pregnant Sprague-Dawley rats (SLAC Laboratory Animal Inc., Shanghai, China) were housed individually and checked daily until delivery. The date of birth of the litter was labeled as postnatal day 0 (PND 0). On PND 1, newborn males were randomly assigned into two groups: (1) the maternally deprived stress group (MD, *N* = 20): these rats were exposed to maternally deprived manipulation from PND1 to PND14; (2) the normal control group (NOR, *N* = 20): these rats were not exposed to any stress conditions. The behavior of rats was measured on the 10th week by a sucrose preference, open field, and forced swimming test. The experiment schedule is shown in Figure 1. The experimental animal protocol was approved by the Animal Ethics Committee of Central South University. All rats were housed with water and food available *ad libitum* on a 12 h light/dark cycle (lights on 7:00–19:00 h).

### Maternal Deprivation (MD)

The MD paradigm was designed as previously described (18). Briefly, litters were deprived from dams for 6 h daily from PND 1 to PND 14 (the deprivations assigned at 9:00–15:00). To block communication among littermates, pups were placed individually in a single cell (8 × 8 × 14 cm for each cell) and covered with dry sawdust. At the end of the deprivation period, litters were returned to their maternal cages. All experiments were carried out in a temperature-controlled room (30°C).

### Open Field Test (OFT)

The open-field test was conducted within a rectangular area (50 × 83 × 56 cm) as previously described (18). At the start of the test, animals were placed at the center of the arena and were allowed to crawl freely. The behavior of rodents was monitored for 5 min, by a video camera mounted on the ceiling above the center of the arena (Ethovision 1.50, Noldus IT, Wageningen, Netherlands). The total distance, vertical counts, percentage of central distance, and fecal pellets were recorded by the computerized tracking system to assess locomotor activity, exploration, and anxiety levels, respectively. After each trial, the box was thoroughly cleaned with 75% ethanol.

### Sucrose Preference Test (SPT)

The SPT was conducted as previously described (18). Rats were kept individually and given free access to two bobbles of water. On the first day, two bottles of tap water were placed in every cage. One bottle of tap water was replaced with a 1.5% sucrose solution on the second day. On the third day, rats were deprived of water for 23-h, and then a bottle of 1.5% sucrose water and a bottle of tap water were given to the rats at a random location in the cage for the last 1-h. The consumption amount of total water and a 1.5% sucrose agent were determined in the last 1-h. The sucrose preference rate was calculated according to the following equation: sucrose preference rate = sucrose intake (g)/[(sucrose intake (g) + tap water intake (g)].

### Forced Swimming Test (FST)

Rats were placed in an open cylindrical container (35 × 32 cm) with water of 29 cm depth and a temperature of 24 ± 1°C, and the experiment was conducted as previously described (18). There was a 15-min practice round before the day of the test. At the same time on the following day, rats were placed in the swimming container individually for a 6-min test. The activities of rats were record by a video camera. When the rat floated without struggling and kept its head above the water, the time spent was defined as the immobility time. The immobility time was recorded in the last 5 min in a 6-min test to assess behavioral despair.

### Enzyme-Linked Immunosorbent Assay (ELISA)

Animals were euthanized with an overdose of pentobarbital on the next day of the behavioral test. Blood was collected into EDTA (ethylenediaminetetraacetic acid) tubes via cardiac puncture under deep anesthesia. The plasma was collected by centrifugation of the blood at 1,500 rpm for 5 min at 4°C and kept at −80°C until testing. The concentration of CORT, DA, and the GDNF in plasma was detected using a Corticosterone EIA Kit (Cayman, Germany), Rat dopamine, DA ELISA kit (R&D, USA) and a Rat GDNF ELISA kit (Sigma, USA) following the manufacturers manual instructions, respectively.

### Real-Time Reverse Transcription Quantitative PCR (qRT-PCR)

According to the rat brain in stereotaxic coordinates, the whole ventral tegmental area (VTA) tissue was immediately collected after blood collection. The total RNA was isolated from the dissected brain tissue according to the standard Trizol (Life Technologies) protocol. q*RT-PCR* was performed as previously described (18). The sequencing primers were ttcaagccaccatcaaaagac and gtagcccaaacccaagtcagt for the GDNF, cacccgcgagtacaaccttc (Forward) and cccatacccaccatcacacc (reverse) for β-actin. The data analysis was performed using the comparative ΔΔCT method. β-actin mRNAs were used as internal control.

### Western Blot

The VTA tissues were homogenized in ice-cold homogenization buffer containing protein and phosphatase inhibitors and a Western blot was performed as previously described (18). Antibodies for the GDNF protein were purchased from Abcam (Cambridge, MA, USA). To control for loading efficiency, the blots were stripped and reprobed with a β-actin antibody. Proteins were normalized to β-actin.

### DNA Isolation and DNA Methylation Analysis

Genomic DNA was isolated from the VTA tissues using a proteinase K/phenol-chloroform extraction method and dissolved in TE buffer. CpG islands in the promoter of the GDNF gene were selected: (1) 200 bp minimum in length; (2) 50% or higher GC content; and (3) 0.60 or higher ratio of observed/expected dinucleotides. Two regions including CpG islands were finally selected and sequenced. Both CpG islands are highly conserved in mice, rats, and humans. BiSulfte Amplicon sequencing PCR was used for quantitative methylation analysis. The methylation level at each CpG site was calculated as the percentage of the methylated cytosines over the total tested cytosines. The average methylation level was calculated.

### Statistical Analysis

Data were analyzed using the statistical software SPSS 17.0 and expressed as mean ± S.D. The Students' *t-*test was used to detect a statistical significance between two groups. Correlations between biomarkers and behavioral indexes were analyzed using the Pearson correlation. A *p* < 0.05 was considered as significant.

## Results

### The Long-Term Effect of Maternal Deprivation on Rats' Behaviors in Adulthood

**Figure 2:**
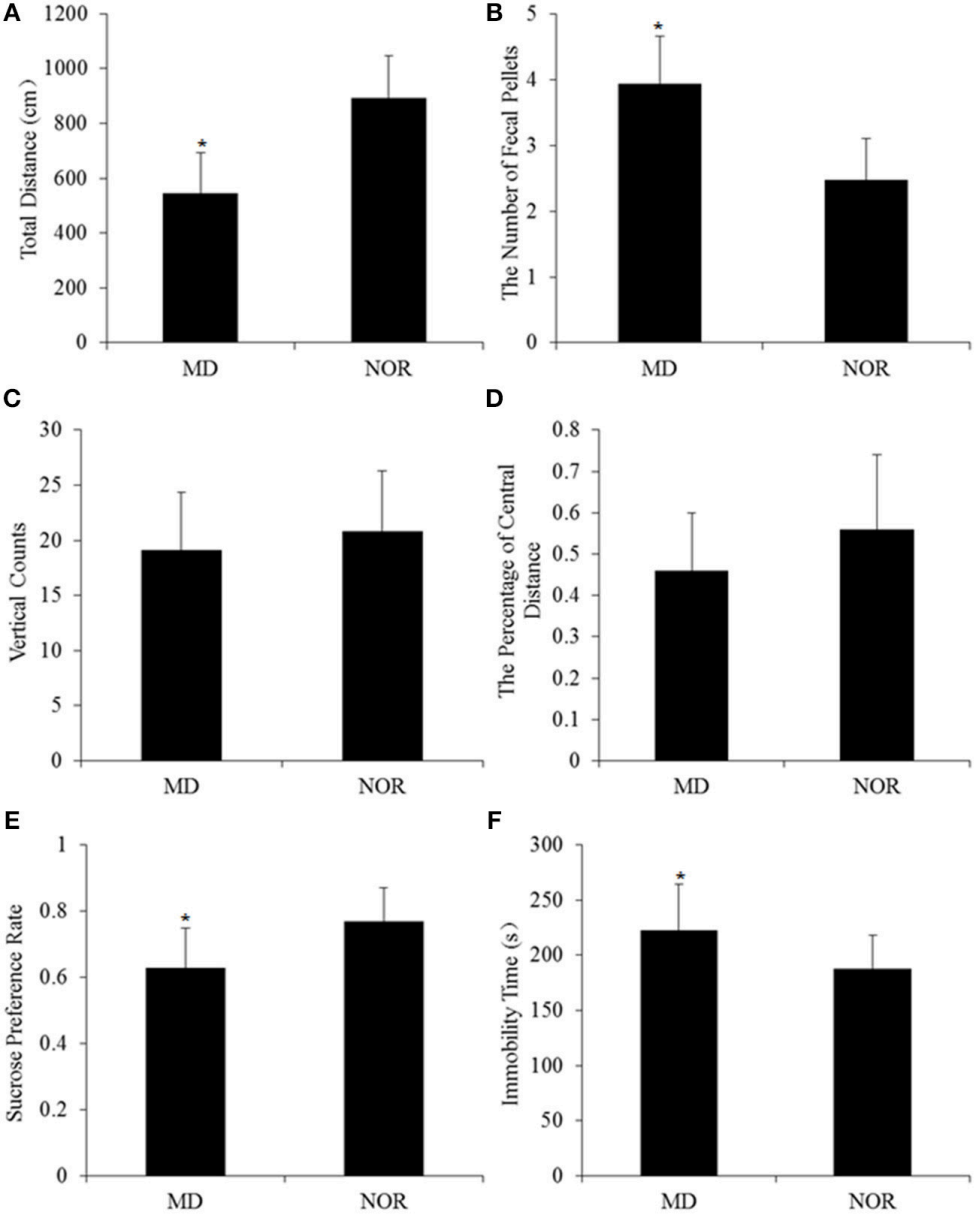
Maternal deprivation (MD)-induced behavioral changes in adult rats. **(A)** Total Distance in the open field test. **(B)** The number of fecal pellets in the open field test. **(C)** Vertical counts in the open field test. **(D)** The percentage of central distance in the open field test. **(E)** Sucrose preference rate in the sucrose preference test. **(F)** Immobility time in the forced swimming test. *p < 0.05 vs. the normal control (NOR) group.

In open field test, the total distance was significantly shorter in MD rats than in NOR rats (*t* = −3.75, *p* = 0.001; Figure 2A). The number of fecal pellets in MD rats was significantly more than in NOR rats (*t* = 2.34, *p* = 0.03; Figure 2B). While there was no significant difference of vertical counts (*t* = −0.63, *p* = 0.54; Figure 2C) and the percentage of central distance (*t* = −1.42, *p* = 0.17; Figure 2D) between the MD group and the NOR group.

In the sucrose preference test, the sucrose preference rate was significantly lower in MD rats than in NOR rats (*t* = −3.45, *p* = 0.002; Figure 2E).

In the forced swimming test, MD rats showed significantly longer immobility time than NOR rats (*t* = 2.53, *p* = 0.02; Figure 2F).

### The Long-Term Effect of Maternal Deprivation on CORT, DA, and GDNF Level in Plasma of Adult Rats

**Figure 3:**
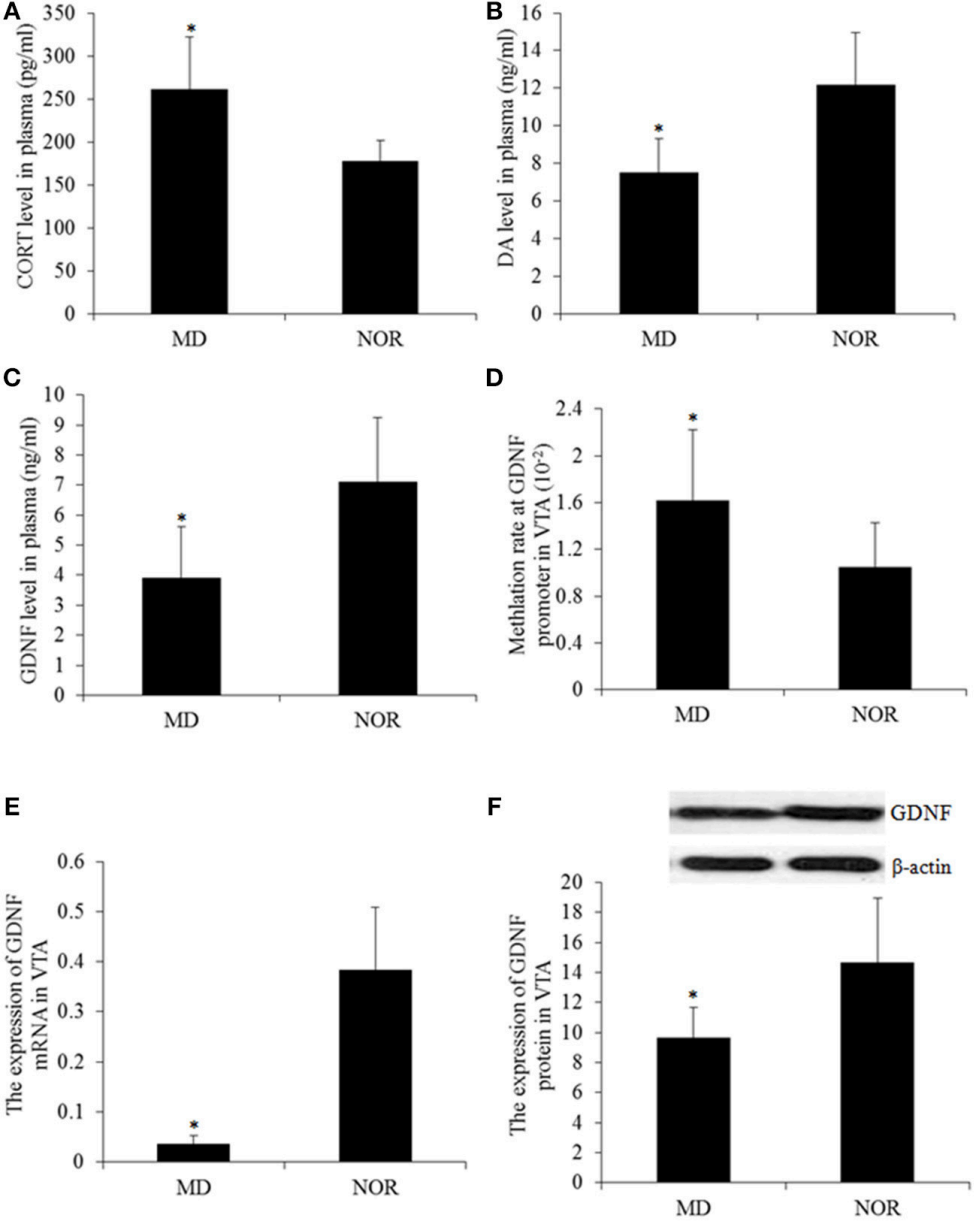
Corticosterone (CORT), Dopamine (DA), and the Glial cell line-derived neurotrophic factor (GDNF) gene expression and methylation. **(A)** Plasma CORT concentration. **(B)** Plasma DA concentration. **(D)** Plasma GDNF concentration. **(D)** The total methylation level at the GDNF promoter in the ventral tegmental area (VTA) tissue. **(E)** The GDNF mRNA level in the VTA tissue. **(F)** Western blot of the GDNF expression in the VTA tissues. *p < 0.05 vs. the NOR group.

The concentration of the plasma CORT in MD rats was significantly higher than that in NOR rats (*t* = 3.17, *p* = 0.01; Figure 3A). While compared with the NOR group, there were significantly lower levels of DA (*t* = −3.45, *p* = 0.006; Figure 3B) and the GDNF (*t* = −2.27, *p* = 0.047; Figure 3C) in the plasma of the MD group rats.

### The Long-Term Effect of Maternal Deprivation on DNA Methylation of the GDNF Gene Promotor, the GDNF mRNA, and Protein in the VTA of Adult Rats

The methylation levels of CpG sites within the GDNF promoter were measured and the results revealed a significantly higher percentage of methylated clones in the VTA of MD rats compared with NOR rats (*t* = 6.55, *p* < 0.001; Figure 3D) Concomitantly, The expression of the GDNF mRNA (*t* = −3.81, *p* = 0.003; Figure 3E) and protein (*t* = −2.61, *p* = 0.026; Figure 3F) in the VTA of MD rats were both significantly lower than that in NOR rats. Furthermore, the GDNF mRNA expression is negatively correlated with the methylation at the gene promoter (*r* = −0.64, *p* < 0.05).

### The Correlation Between Behavior Indexes and Biomarkers

The total distance significantly correlated with the plasma DA level (*r* = 0.74, *p* < 0.01), the plasma GDNF level (*r* = 0.61, *p* < 0.05), the DNA methylation level at the GDNF promoter (*r* = −0.66, *p* < 0.05) and the GDNF mRNA level (*r* = 0.769, *p* < 0.01) in the VTA. The number of fecal pellets showed a significant correlation with the plasma CORT (*r* = −0.75, *p* < 0.01). The sucrose preference rate was significantly correlated with the plasma DA level (*r* = 0.65, *p* < 0.05), the DNA methylation level at the GDNF promoter (*r* = −0.67, *p* < 0.05) and the GDNF mRNA level (*r* = 0.71, *p* < 0.05) in the VTA. Immobility time showed a significant correlation with the plasma DA level (*r* = −0.58, *p* < 0.05), the plasma GDNF level (*r* = −0.61, *p* < 0.05) and the GDNF mRNA level (*r* = −0.68, *p* < 0.05) in the VTA (Table 1).

**Table 1:** Correlation between biomarkers and behavioral indexes (r).

		**Total distance**	**Fecal pellets**	**Sucrose preference rate**	**Immobility time**
In plasma	CORT	−0.09	0.75^**^	−0.38	0.42
	DA	0.74^**^	−0.19	0.65^*^	−0.58^*^
	GDNF	0.61^*^	−0.20	−0.09	−0.61^*^
In VTA	Methylation level of GDNF promoter	−0.66^*^	0.38	−0.67^*^	0.45
	GDNF mRNA level	0.77^**^	−0.16	0.71^*^	−0.68^*^
	GDNF Protein level	0.54	−0.24	0.16	−0.41

* *p < 0.05*,

** *p < 0.01*.

## Discussion

Anhedonia is one of the core symptoms of MDD (19), and was examined in this study using a sucrose preference test. Results showed that MD significantly decreased the sucrose preference rate. The behavioral despair in MD rats were reflected by the increased immobility time during the forced swimming test (20). Our findings suggest that early life maternal deprivation has a long-term effect on rodent behaviors and induce depressive-like behaviors in adult rats. The locomotor activity, exploration, and anxiety level were reflected by the total distance, vertical counts, percentage of central distance and fecal pellets in the open field test, respectively. In this study, early life maternal deprived rats showed a significantly shorter total distance and more fecal pellets. The results indicate that MD increased anxiety-like behaviors and decreased locomotor activity, which is consistent with the psychomotor retardation and anxiety in depressive patients. Altogether, these results indicate that early life maternal deprivation has a long-term effect on rats' behaviors and induce depression- and anxiety-like behavior in adulthood, which is similar as our previous studies (3, 13, 21).

Previous studies have shown that the hypothalamic-pituitary-adrenal (HPA) axis is involved in individual experiences of psychological stress (22). Corticosterone (CORT), as a glucocorticoid, is widely used to reflect an individual stress response. In this study, rats exposed to early life maternal deprivation stress still had a significantly higher level of plasma CORT in adulthood, indicating that early life stress can induce long-term high levels of CORT. This result is consistent with previous studies, that indicate that early life adversity affects the individual HPA axis and induces an aberrant stress coping style in individuals. Furthermore, the plasma CORT level showed a significant correlation with anxiety-like behaviors, which suggests that CORT levels not only reflect individuals in a status of stress, but also reflects an individual's high level of anxiety.

Dopamine is an important neurotransmitter in the brain, which is closely related to stress-induced depression. Many studies have found that early life stress can induce abnormal changes in the dopaminergic system (18, 23). In the current study, maternal deprivation stress significantly reduced plasma dopamine concentration. When the dopaminergic system in the CNS is damaged, such as the dopaminergic neuron, the secretion of the dopamine and the blood that enters through the blood-brain barrier both decrease. Therefore, low levels of dopamine in the blood reflect the abnormality of the central dopamine system to some extent. In addition, the level of plasma dopamine was significantly correlated with the rate of sucrose preference, immobility time and the total distance in MD rats, suggesting that maternal deprivation stress-induced depression-like behaviors may be closely related to the inactivation of the dopamine system.

It has been found that the GDNF plays a crucial role in the nutritional support of central dopaminergic neurons and can promote the development and repair of dopaminergic neurons (24). Human and animal studies have shown that high expression of the GDNF can promote the growth of transplanted dopaminergic neurons and alleviate the damage of the dopaminergic system induced by psychological stress (25, 26). In this study, maternal deprivation stress down-regulated the plasma GDNF level, and the expression of the GDNF mRNA and protein in the VTA. The expression of the GDNF mRNA in the VTA was significantly correlated with anhedonia, despair, and locomotor activity, suggesting that the GDNF is associated with maternal deprivation-induced depression-like behaviors. A recent study has demonstrated that the GDNF is important for the pathogenesis of depression, such as the decrease of the GDNF protein and mRNA expression in the serum and hippocampus of depressive individuals (27). According to previous studies on the effect of the GDNF on dopaminergic neurons, it was suggested that early life stress down-regulates the expression of the GDNF gene in the VTA, subsequently impair the nutritional effect of the GDNF on the growth and function maintenance of dopaminergic neurons, destroy the repairing effect of the GDNF on the damaged dopaminergic neurons, which finally leads to the onset of depression-like behavior.

Increasing evidence suggests that aberrant transcription regulation, such as the epigenetic regulation of some critical genes in the brain, is a key component in the pathophysiology of depression (28, 29). DNA methylation of genes could trigger the development of depression symptoms in response to psychological stress (30–33). The results in this study showed that MD rats had a high level of DNA methylation at the promoter of the GDNF gene in the VTA which may down-regulate the expression of the GDNF gene. Furthermore, the methylation level of the GDNF gene correlated with anhedonia and locomotor activity, suggesting that a high level of DNA methylation in the GDNF gene in the VTA, may be one of the regulatory mechanisms of maternal deprivation-induced adult depression in rats. A study by Uchida et al. (17) showed that epigenetic regulation of the GDNF promoter in the NAc is associated with the susceptibility and the adaptation responses to chronic stress. Collectively these data indicate that long-term changes in the GDNF expression, induced by early life maternal deprivation, may be the underlying factor that increases the probability of developing psychopathology later in life.

In conclusion, up-regulation of the DNA methylation at the GDNF gene promotor and the subsequent down-regulation of the GDNF gene expression in the VTA, may be involved in the development of depression-like behaviors in rats experiencing maternal deprivation in early life.

## Author Contributions

YZ, LW, XW, and YW performed the experiments, data analysis, and prepared the manuscript. YZ, LW, XZ, and CL designed the study, revised the manuscript, and approved the final version.

### Conflict of Interest Statement

The authors declare that the research was conducted in the absence of any commercial or financial relationships that could be construed as a potential conflict of interest.

## Abbreviations

CNS: central nervous system
CORT: corticosterone
DA: dopamine
ELISA: Enzyme-linked immunosorbent assay
FST: forced swimming test
GDNF: glial cell line-derived neurotrophic factor
HPA: hypothalamic-pituitary-adrenal
MDD: major depressive disorder
MD: maternal deprivation group
OFT: open Field Test
PND: postnatal day
SD: Sprague-Dawley
SPT: sucrose preference test
VTA: ventral tegmental area.
